# A Porcine *Ex Vivo* Lung Perfusion Model To Investigate Bacterial Pathogenesis

**DOI:** 10.1128/mBio.02802-19

**Published:** 2019-12-03

**Authors:** Amy Dumigan, Marianne Fitzgerald, Joana Sá-Pessoa Graca Santos, Umar Hamid, Cecilia M. O’Kane, Danny F. McAuley, Jose A. Bengoechea

**Affiliations:** a Wellcome-Wolfson Institute for Experimental Medicine, School of Medicine, Dentistry and Biomedical Sciences, Queen’s University Belfast, Belfast, United Kingdom; Virginia-Maryland Regional College of Veterinary Medicine; Emory University School of Medicine

**Keywords:** *Klebsiella*, lung infection, macrophages, pig

## Abstract

The use of animal infection models is essential to understand microbial pathogenesis and to develop and test treatments. Insects and two-dimensional (2D) and 3D tissue models are increasingly being used as surrogates for mammalian models. However, there are concerns about whether these models recapitulate the complexity of host-pathogen interactions. In this study, we developed the *ex vivo* lung perfusion (EVLP) model of infection using porcine lungs to investigate Klebsiella pneumoniae-triggered pneumonia as a model of respiratory infections. The porcine EVLP model recapitulates features of K. pneumoniae-induced pneumonia lung injury. This model is also useful to assess the pathogenic potential of K. pneumoniae, as we observed that the attenuated *Klebsiella* capsule mutant strain caused less pathological tissue damage with a concomitant decrease in the bacterial burden compared to that in lungs infected with the wild type. The porcine EVLP model allows assessment of inflammatory responses following infection; similar to the case with the mouse pneumonia model, we observed an increase of *il-10* in the lungs infected with the wild type and an increase of *ifn-γ* in lungs infected with the capsule mutant. This model also allows monitoring of phenotypes at the single-cell level. Wild-type K. pneumoniae skews macrophages toward an M2-like state. *In vitro* experiments probing pig bone marrow-derived macrophages uncovered the role for the M2 transcriptional factor STAT6 and that *Klebsiella*-induced *il-10* expression is controlled by p38 and extracellular signal-regulated kinase (ERK). *Klebsiella*-induced macrophage polarization is dependent on the capsule. Together, the findings of this study support the utility of the EVLP model using pig lungs as a platform to investigate the infection biology of respiratory pathogens.

## Abstract (precis)

The implementation of infection models that approximate human disease is essential to understand infections and for testing new therapies before they enter into clinical stages. Rodents are used in most preclinical studies, although the differences between mice and humans have fueled the conclusion that murine studies are unreliable predictors of human outcomes. In this study, we have developed a whole-lung porcine model of infection using the *ex vivo* lung perfusion (EVLP) system established to recondition human lungs for transplant. As a proof of principle, we provide evidence demonstrating that infection of the porcine EVLP with the human pathogen Klebsiella pneumoniae recapitulates the known features of *Klebsiella*-triggered pneumonia. Moreover, our data revealed that the porcine EVLP model is useful to reveal features of the virulence of K. pneumoniae, including the manipulation of immune cells. Together, the findings of this study support the utility of the EVLP model using pig lungs as a surrogate host for assessing respiratory infections.

## INTRODUCTION

The use of animal infection models is essential to determine basic physiological principles and disease pathogenicity, to identify virulence factors, and to develop and test treatment strategies (1). The vast majority of immunology studies employ murine models, owing to the availability of transgenic knockouts, reagents, and established protocols. Therefore, our current knowledge of the murine immune system far exceeds that of any other species. However, murine models have several limitations: there are significant differences between mice and humans in immune system development, activation, and response to challenge (2). Indeed, mice share <10% genetic homology with the human immune system (3). The increasing costs related to animal husbandry, making large-scale infection experiments expensive, and growing social concerns on the use of mice for biomedical experimentation despite the extensive and comprehensive animal welfare regulations in place are additional drawbacks.

To circumvent these issues, alternative models of infection are being explored. Insects, including Drosophila melanogaster and Galleria mellonella (4), and the fish Danio rerio (5) are increasingly being used to investigate host-pathogen interactions. These models have proved successful in identifying virulence factors and to model features of the interaction between pathogens and the innate immune system. However, there are still concerns about whether these infection models recapitulate the complex interactions between several immune cells, cytokines and chemokines and other soluble factors, such as complement, and pathogens.

To address these issues, new infection models have been developed, including two-dimensional (2D) polarized epithelium and 3D organoids of different tissues. These models still fall short of recapitulating the complex interactions between different cells as well as the structure of the organ. This study was initiated to establish a new infection model to investigate respiratory infections, the *ex vivo* lung perfusion (EVLP) model of infection using porcine lungs. Next to nonhuman primates, the domestic pig (Sus scrofa *domesticus*) has the closest genome and protein sequences to those of humans (6). Like humans, pigs are omnivores and have adaptive and innate immune systems, and pigs and humans share similar anatomy and physiology. Indeed, the porcine immune system is functionally more similar to the human immune system than that of mice, sharing >80% genetic homology (6). Notably, it is believed that experiments with pigs have more predictive therapeutic value than research carried out with rodents (7). The model developed in this study facilitates the investigation of pathogen infection biology in a whole porcine lung receiving ventilation and perfusion in real time. This allows the investigation of the spatial distribution of infection, innate immune cell recruitment and activation, and histopathological changes. As a proof of concept, we have investigated whether this model recapitulates key features of Klebsiella pneumoniae-induced pneumonia.

K. pneumoniae is an important cause of nosocomial and community-acquired pneumonia. *Klebsiella* can readily spread between hospital patients, with devastating results in immunocompromised individuals and with mortality rates between 25 and 60% depending on the underlying condition (8). K. pneumoniae has been singled out by the World Health Organization as an urgent threat to human health due to the increasing isolation of multidrug-resistant strains. A wealth of evidence obtained using the pneumonia mouse model demonstrates that clearance of K. pneumoniae relies on the activation of an inflammatory response which includes the activation of type I interferon (IFN)-controlled host defense responses (9, 10). Several studies have demonstrated the importance of alveolar macrophages and inflammatory monocytes in the containment and clearance of K. pneumoniae in the lungs (11–14). Conversely, this may suggest that a signature of K. pneumoniae infection biology is the attenuation of inflammatory responses and the subversion of macrophage-governed antimicrobial functions. Indeed, we and others have shown that in sharp contrast to wild-type strains, attenuated mutant *Klebsiella* strains activate an inflammatory program, ultimately favoring their clearance (15–18). Furthermore, we have recently demonstrated that K. pneumoniae is able to survive intracellularly in mouse and human macrophages by preventing the fusion of lysosomes with the *Klebsiella*-containing vacuoles (19).

Here we report that the porcine EVLP infection model recapitulates key features of *Klebsiella*-triggered pneumonia. We present data showing that this model is also useful to assess the pathogenic potential of K. pneumoniae, as we observed that the attenuated *Klebsiella* capsule mutant strain caused less pathological damage to the tissue with a concomitant decrease in the bacterial burden compared to that in lung infected with the wild-type strain. Finally, we present evidence demonstrating that K. pneumoniae skews macrophage polarization following infection in a STAT6-dependent manner.

## RESULTS

### *Ex vivo* lung porcine model of infection.

In this study, we have developed a whole-lung porcine model of infection using the established EVLP model developed to recondition human lungs that were marginal at meeting the lung retrieval criteria with the view to increase the lung donor pool for transplant (20). In this work, we have used one of the four commercially available clinical grade devices for EVLP, the Vivoline LS1 system. We selected a livestock porcine breed, as such breeds are readily available and have been shown to better mimic animal variation reflective of human populations than wild breeds (7).

There are a number of essential details to consider when setting up the porcine EVLP model. The quality of the organ is an essential factor, and researchers should carefully assess whether there are any macroscopic signs of damage/infection. The model uses 200 ml of autologous whole blood, which acts as a reservoir for immune cell recruitment and should be taken prior to lung retrieval. Lungs are removed from the pig and flushed with medium through the pulmonary artery to remove blood. This is essential to avoid clotting. Lungs were then transferred to a sterile plastic bag on ice for transport to the laboratory.

**FIG 1:**
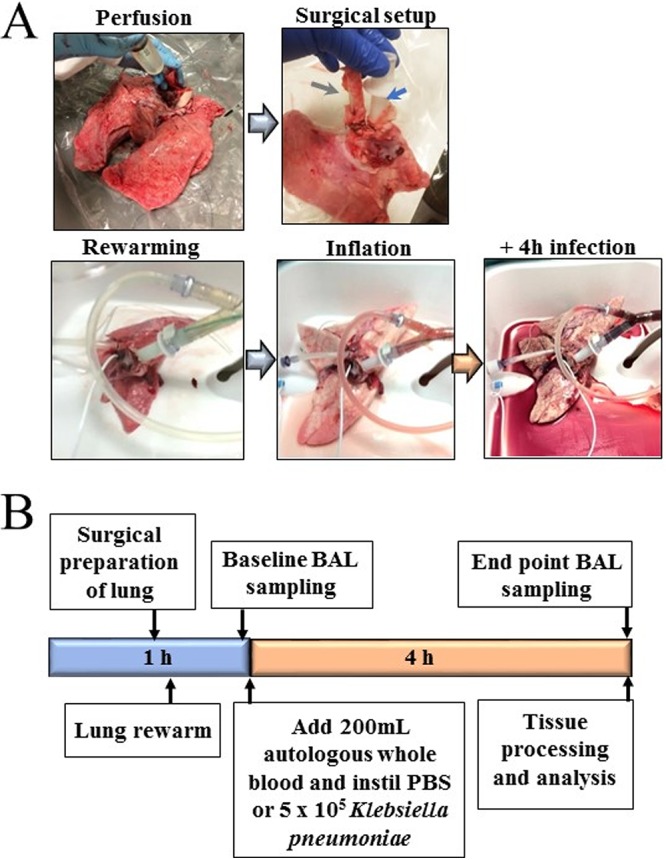
The porcine EVLP infection model. (A) Images of lung during experimental process. The green arrow indicates LS1 ET tube in main bronchus, and the blue arrow indicates a catheter placed in the pulmonary artery of the left lung. (B) Schematic describing the experimental design.

Unlike humans, pigs have an additional bronchus emerging from the trachea supplying the cranial lobe of the right lung (21). Therefore, only left lungs were used in this investigation, as they are immediately suitable for use on the LS1 system. However, preliminary experimentation revealed that by occluding the second bronchus on the right lung with a purse string suture, right lungs can also be used. A cannula is placed in the pulmonary artery and secured with surgical sutures. An LS1 endobronchial tube is placed in the main bronchus and also secured with sutures. To avoid inducing tissue damage and abnormal inflammatory responses, the lungs should be warmed before any other manipulation, and the pulmonary perfusate and ventilation should be carefully managed in a gradual way. The lung is then connected to the LS1 system with perfusion but no ventilation and allowed to warm, ensuring that the shunt is open at this time. Once lung has reached 37°C, lung is inflated by hand using a bag-valve positive-pressure ventilation assist device (Ambu-bag). The lung is then connected to a ventilator and receives 10 cm H_2_O of continuous positive airway pressure (CPAP) with a mixture of 95% oxygen and 5% CO_2_ (Fig. 1A). A detailed description of the preparation of the lungs and setup of the EVLP model is provided in Materials and Methods. A schematic of a typical experimental design can be seen in Fig. 1B.

Preliminary experiments were carried to optimize the inoculum of K. pneumoniae 52.145 (here referred to as Kp52145) and the time of infection based on macroscopic changes to the lungs. This K. pneumoniae strain clusters with those strains frequently associated with human infection and encodes all virulence functions significantly associated with invasive community-acquired disease in humans (22, 23). The virulence of this strain has been tested in several infection models, including mice, rats, G. mellonella, and Dyctiostelium discoideum (24–27). An inoculum of 5 × 10^5^ CFU and 4-h infection period were selected in this study based on assessment of macroscopic damage of lungs during infection. Once lung had been warmed to 37°C, a catheter was inserted into the caudal lobe of the lung and a baseline bronchoalveolar lavage (BAL) carried out. With the catheter still in place, lungs received 5 ml of sterile phosphate-buffered saline (PBS) or were inoculated with the bacterial inoculum. After 4 h of infection, a second BAL sample was collected and assessed for immune cell recruitment and protein levels. At the experimental endpoint, tissue samples were collected from the cranial, middle, and caudal areas of the lung (see Fig. S1 in the supplemental material) and analyzed for edema, bacterial CFU, and histology. Single samples were taken from the caudal lobe to assess immune cell recruitment (using flow cytometry) and gene transcription via real-time qPCR (RT-qPCR).

10.1128/mBio.02802-19.1 FIG S1 Regions for sample selection from porcine EVLP lung. The image identifies cranial, middle, and caudal regions for tissue sample collection. Download FIG S1, PDF file, 0.01 MB. Copyright © 2019 Dumigan et al. 2019 Dumigan et al. This content is distributed under the terms of the Creative Commons Attribution 4.0 International license.

### Tissue damage in the porcine EVLP model reflects hallmarks of *Klebsiella*-induced human pathology.

**FIG 2:**
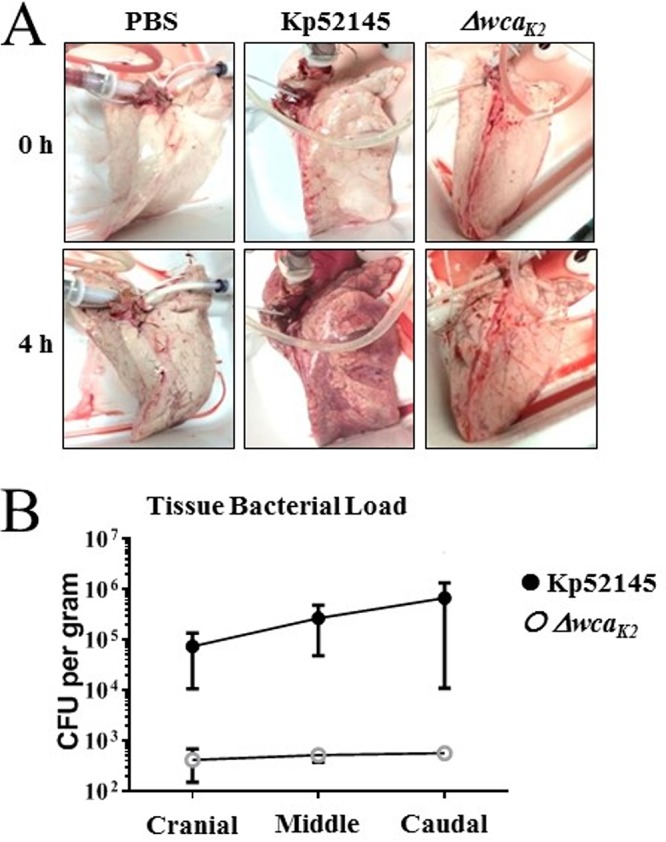
Infection of whole lungs with K. pneumoniae induces lung damage. (A) Images of macroscopic damage of the lungs before and after infection with K. pneumoniae 52.145 (Kp52145) and the isogenic *cps* mutant, strain 52145-Δ*wca_K2_*. (B) Bacterial load across different sections of the lungs infected with Kp52145 and strain 52145-Δ*wca_K2_*. Values are presented as the means ± SEM from three independent experiments.

Infection of porcine lungs with Kp52145 led to macroscopic damage after 4 h, in stark contrast to findings for PBS mock-infected lungs (Fig. 2A). K. pneumoniae capsule polysaccharide (CPS) is a well-characterized virulence factor of *Klebsiella* (28, 29)*. cps* mutant strains are avirulent in mammalian and nonmammalian models of disease (24, 25, 28, 29). To determine the sensitivity of the porcine model, lungs were infected with 5 × 10^5^ CFU of strain Kp52145-Δ*wca_K2_* in 5 ml of PBS. As shown in Fig. 2A, infection with the *cps* mutant resulted in limited macroscopic damage, suggesting that *cps* also plays a crucial role in infection biology of K. pneumoniae in the porcine EVLP infection model.

To establish whether the macroscopic damage in the lungs infected with the wild-type strain was associated with higher bacterial burden in the tissue, samples were collected across the lung, as shown in Fig. S1, and homogenized, and the number of CFU per gram of tissue was determined. Indeed, the bacterial burden was 3 logs higher in lungs infected with the wild-type strain than in those infected with the *cps* mutant (Fig. 2B). Interestingly, despite the inoculum being introduced in the caudal lobe, bacterial burden was homogenously distributed across the lung (Fig. 2B).

**FIG 3:**
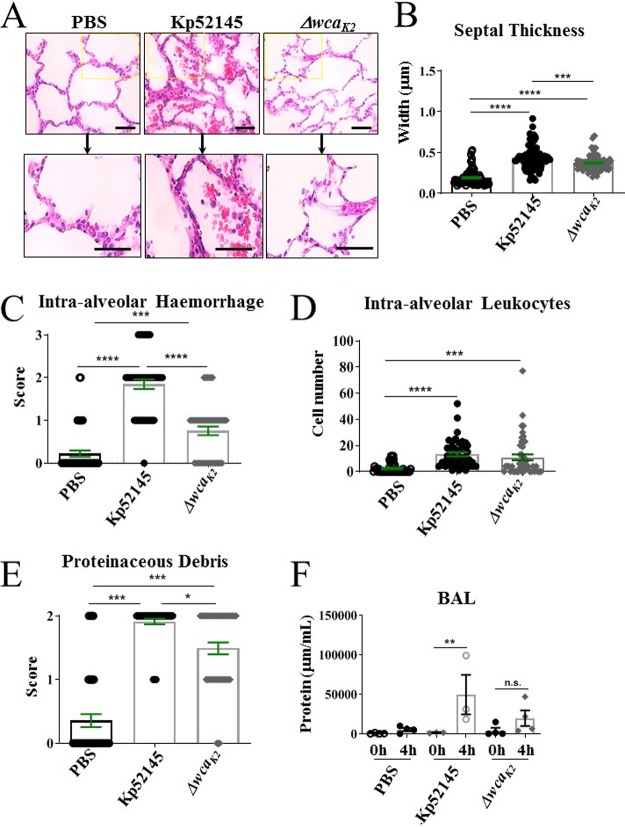
Porcine EVLP model recapitulates clinical hallmarks of K. pneumoniae-induced pneumonia. (A) Hematoxylin and eosin staining of porcine lung samples (magnification, ×400) from lungs mock infected (PBS) or infected with Kp52145 and strain 52145-Δ*wca_K2_*. (B) Alveolar septal thickness was measured using ImageJ software. Each dot represents an average of three alveolar thicknesses per image, corresponding to three sections per lung across three experimental replicates from lungs mock infected (PBS) or infected with Kp52145 and strain 52145-Δ*wca_K2_*. (C) Intra-alveolar hemorrhage was scored per image whereby 0, 1, 2, and 3 represent none, mild, moderate, and severe levels of red blood corpuscles within the alveolar space from lungs mock infected (PBS) or infected with Kp52145 and strain 52145-Δ*wca_K2_*. (D) Number of nucleated cells evident in the alveolar space per image from lungs mock infected (PBS) or infected with Kp52145 and strain 52145-Δ*wca_K2_*. (E) Scoring of proteinaceous debris in the alveolar space from lungs mock infected (PBS) or infected with Kp52145 and strain 52145-Δ*wca_K2_*. (F) Protein levels at baseline and endpoint BAL samples from whole lungs mock infected (PBS) or infected with Kp52145 and strain 52145-Δ*wca_K2_*. Statistical analysis was carried out using one-way ANOVA with Bonferroni correction. Error bars indicate SEM.

Histological analysis of porcine tissues was carried out based on parameters of acute respiratory distress syndrome (ARDS) in animal models as defined by the American Thoracic Society. Pathogenic hallmarks of lung injury include thickening of alveolar septa and infiltration of proteinaceous debris, red blood cells (hemorrhage), and immune cells, including neutrophils, into the alveolar space (neutrophilic alveolitis) (30). Analysis of lung sections stained with hematoxylin-eosin revealed signs of injury in the infected lungs, although injury was more severe in those lungs infected with Kp52145 (Fig. 3A). This was further confirmed by analysis of alveolar septal thickness (Fig. 3B). This measurement revealed significant thickening of alveolar septal membranes in lungs infected with Kp52145. Infection with the *cps* mutant strain induced significantly enhanced alveolar septal thickening compared to that of PBS controls; however, this damage was significantly reduced compared to that of Kp52145-infected lungs (Fig. 3B). One hallmark of K. pneumoniae-triggered necrotizing pneumonia is the presence of cherry red (blood streaked) sputum, i.e., hemorrhage. Hemorrhage was clearly evident both macroscopically (Fig. 2A) and microscopically (Fig. 3A) in lungs infected with Kp52145 and significantly reduced in the lungs infected with the *cps* mutant. The presence of intra-alveolar hemorrhage was assigned a score of 0, 1, 2, or 3 based on a semiquantitative assessment of none, mild, moderate, or severe. Scoring confirmed significantly enhanced hemorrhage in lungs infected with Kp52145 compared to that in the PBS-mock-infected lungs and the lungs infected with the *cps* mutant (Fig. 3C). Hemorrhage was accompanied by the presence of inflammatory immune cells within the alveolar space. The number of nucleated cells in the alveolar space was quantified, and it was significantly higher in the lungs infected with Kp52145 than in those infected with the *cps* mutant or PBS-mock infected (Fig. 3D). The presence of proteinaceous debris was significantly higher in the infected lungs than in those PBS-mock infected. However, proteinaceous debris was significantly higher in lungs infected with Kp52145 than in those infected with the *cps* mutant (Fig. 3E). Further supporting the idea that infection with Kp52145 was associated with an increase in lung injury, a 35-fold increase in the total levels of BAL protein was found in the lungs infected with the wild-type strain. There were no differences in the total BAL protein between lungs infected with the *cps* mutant and those PBS-mock infected (Fig. 3F). These findings suggest that infection with the wild-type strain affected alveolar epithelial-endothelial barrier function.

Collectively, these findings demonstrate that the porcine EVLP model recapitulates features of K. pneumoniae-induced pneumonia lung injury. Furthermore, our results demonstrate that this model is useful to assess the virulence of K. pneumoniae since the *cps* mutant, known to be attenuated in other infection models (24, 25, 28, 29), was also attenuated in the porcine EVLP model.

### Innate immune cell recruitment in the K. pneumoniae EVLP model.

**FIG 4:**
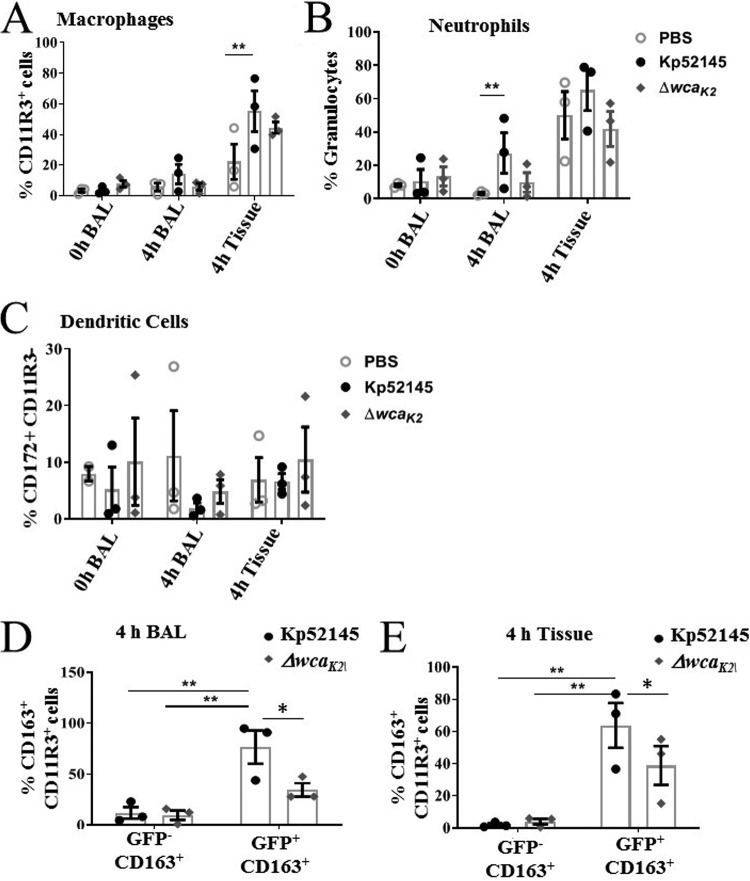
Innate cell recruitment in K. pneumoniae-infected porcine EVLP model. (A) CD11R3^+^ macrophages at baseline (0 h) and endpoint (4 h posttreatment) in BAL samples and tissue from caudal lobes mock infected (PBS) or infected with Kp52145 and strain 52145-Δ*wca_K2_*. (B) Granulocytes at baseline (0 h) and endpoint (4 h posttreatment) in BAL samples and tissue from caudal lobes mock infected (PBS) or infected with Kp52145 and strain 52145-Δ*wca_K2_*. (C) CD172^+^ dendritic cells at baseline (0 h) and endpoint (4 h posttreatment) in BAL samples and tissue from caudal lobes mock infected (PBS) or infected with Kp52145 and strain 52145-Δ*wca_K2_*. (D and E) Percentages of CD11R3^+^ macrophages positive for CD163 expression associated (GFP^+^) or not (GFP^−^) with Kp52145 and strain 52145-Δ*wca_K2_* harboring plasmid pFPV25.1 Cm in BAL fluid (D) and tissue (E). In all panels, values are represented as means ± SEM from three independent experiments. **, *P* < 0.001; *, *P* < 0.05 (determined by unpaired *t* test).

We next sought to investigate the innate immune response to K. pneumoniae infection in the porcine EVLP model. A total of 100 μg of tissue was removed from the caudal lobe and homogenized. Red blood cells were removed from BAL and tissue samples using ammonium chloride-potassium lysis buffer. Samples were then stained for innate immune cells using purified anti-pig antibodies conjugated with fluorophores and analyzed by flow cytometry. CD11R3 has an expression pattern similar to that of the human CD11b marker, being expressed on pig monocytes and alveolar macrophages but not on lymphocytes, red blood cells, or platelets (31, 32), and was used to assess macrophages. Porcine CD172a, a marker of dendritic cells (33), and the porcine specific granulocyte marker clone 6D10 (Bio-Rad) to identify neutrophils were also used (31, 32). Lungs infected with Kp52145 showed an increase in the number of macrophages in tissue (Fig. 4A). Macrophages are presented as percentage single CD11R3^+^ cells (gating strategy and representative dot plots supplied in Fig. S2A). Neutrophils were identified using a porcine granulocyte marker (clone 6D10) in a similar fashion (Fig. S2B) and were shown to be increased in density in 4-h BAL fluid in the Kp52145-infected experimental group (Fig. 4B). No significant change was observed in CD11R3^−^ CD172^+^ dendritic cells (Fig. 4C) (gating strategy described for Fig. S2C). The numbers of macrophages and neutrophils in the tissue and BAL from lungs infected with the *cps* mutant were lower than those found in the wild-type-infected lungs and closer to the number found in PBS-mock-infected lungs (Fig. 4B). Enhanced macrophage and neutrophil recruitment in Kp52145-infected BAL samples and lung tissue, respectively, correlates with injury observed in histological analysis (Fig. 3).

10.1128/mBio.02802-19.2 FIG S2 Innate cell recruitment in K. pneumoniae-infected EVLP porcine model. Gating strategy and representative dot plots for flow cytometric analysis of CD11R3^+^ macrophages (A), neutrophil staining using anti-pig granulocyte marker clone 6D10 (B), and analysis of CD11R3^−^ CD172^+^ dendritic cells (C). Dot plots represent 0-h (baseline) and 4-h-postinfection or mock infection BAL samples and 4-h tissue samples. (D) Gating strategy to identify differential expression of M2 marker CD163 on K. pneumoniae-infected macrophages (CD11R3^+^ GFP^+^ CD163^+^) or noninfected (−) macrophages (CD11R3^+^ GFP^−^ CD163^+^). Download FIG S2, PDF file, 0.3 MB. Copyright © 2019 Dumigan et al. 2019 Dumigan et al. This content is distributed under the terms of the Creative Commons Attribution 4.0 International license.

The presence of bacteria in tissues is associated with macrophage reprogramming (34). M1 (classical) polarization is associated with protection during acute infections, whereas the M2 (alternative) program is linked to the resolution of inflammation and tissue regeneration (34). Therefore, we sought to establish whether K. pneumoniae infection could be linked to a macrophage switch in polarization. To investigate this possibility, we assessed the levels of the known M2 macrophage marker CD163, an iron scavenger receptor, in infected macrophages (35). Infections were carried out with bacteria expressing green fluorescent protein (GFP) to assess CD163 levels in cells with and without associated bacteria. Flow cytometry experiments showed that the levels of CD163 were significantly higher in those macrophages associated with Kp52145 (CD11R3^+^ GFP^+^ CD163^+^) than in those without bacteria (CD11R3^+^ GFP^−^ CD163^+^) (Fig. 4D) (gating strategy can be found in Fig. S2D). Interestingly, when infections were done with the *cps* mutant, the levels of CD163 were significantly lower in macrophages associated with the mutant than in those associated with the wild-type strain (Fig. 4D and E), suggesting that the CPS may contribute to expression of CD163 on macrophages in K. pneumoniae-infected lungs.

### K. pneumoniae-induced inflammation in the porcine EVLP model.

**FIG 5:**
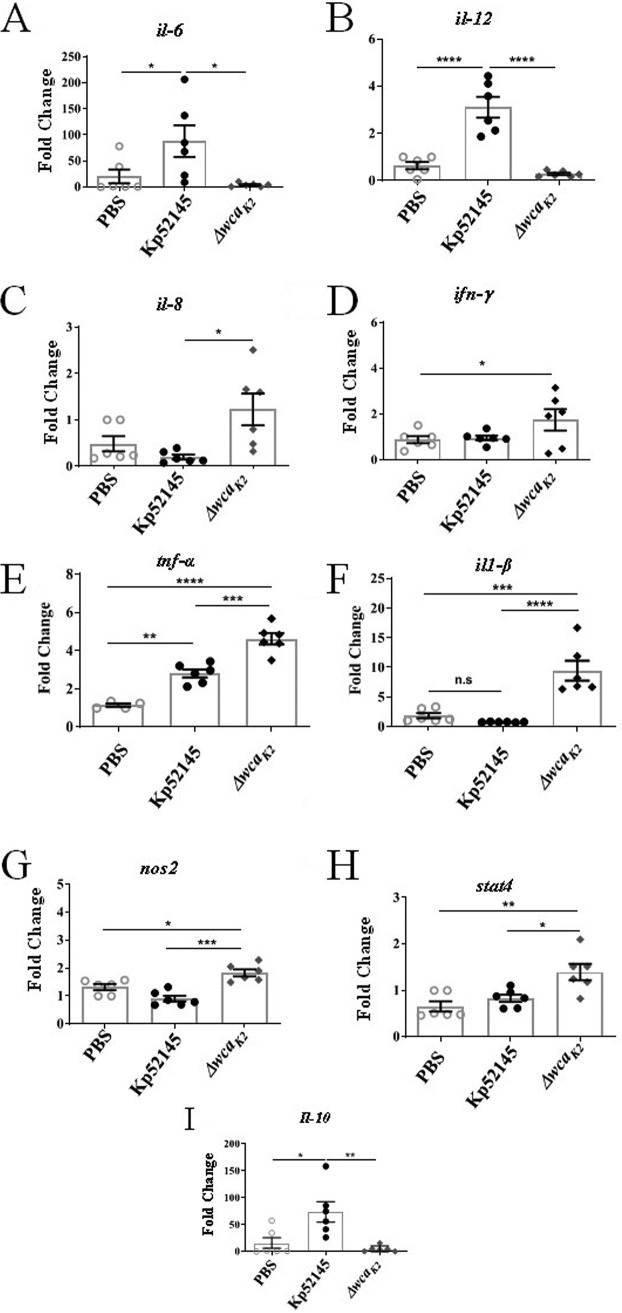
K. pneumoniae-induced inflammation in the porcine EVLP model. mRNA levels in lung tissues mock infected (PBS) or infected with Kp52145 and strain 52145-Δ*wca_K2_* assessed by RT-qPCR. (A) *il-6*; (B) *il-12*; (C) *il-8*; (D) *ifn-γ*; (E) *il-1β*; (F) *tnf-α*; (G) *nos2*; (H) *stat-4*; (I) *il-10*. Values are presented as the means ± SEM from three independent experiments measured in duplicate. Statistical significance for the indicated comparisons was determined using one-way ANOVA with Bonferroni correction.

To further investigate the host response to K. pneumoniae in the porcine EVLP model, we analyzed the expression of several inflammation-associated cytokines and chemokines by RT-qPCR from samples collected from the caudal lobe of lungs. Higher levels of *il-6* and *il-12* were detected in the lungs infected with Kp52145 than in those infected with the *cps* mutant or PBS-mock infected (Fig. 5A and B). In contrast, the levels of *il-8*, *tnf-α*, *il-1β*, and *ifn-γ* were significantly higher in the lungs infected with the *cps* mutant than in those infected with Kp52145 (Fig. 5C to F). Mice deficient in IFN-γ production suffer greater mortality from K. pneumoniae infection (36–39). The higher levels of IFN-γ that are produced during *cps* mutant infection in the EVLP model are likely a result of the high rate of clearance of the capsule mutant strain.

The expression levels of *nos2* and *stat4* were also significantly higher in the lungs infected with the *cps* mutant than in those infected with the wild-type strain, which were similar to those in lungs PBS-mock infected (Fig. 5G and H). These markers have been associated with M1 polarized macrophages (35). We observed a significant increase in the levels of the anti-inflammatory cytokine *il-10* only in the lungs infected with Kp52145 (Fig. 5I). A similar observation has been reported previously for the mouse pneumonia model (40, 41). Notably, enhanced production of *il-10* is one of the features of M2 polarized macrophages, which are associated with resolution of inflammation (34, 35, 42).

Collectively, these findings demonstrate that the porcine EVLP model is useful to assess inflammatory responses following infection. By assessing *Klebsiella*-induced responses, our results imply that wild-type K. pneumoniae may modulate macrophage polarization toward the M2 state.

### K. pneumoniae drives macrophage polarization in a STAT6-dependent manner.

**FIG 6:**
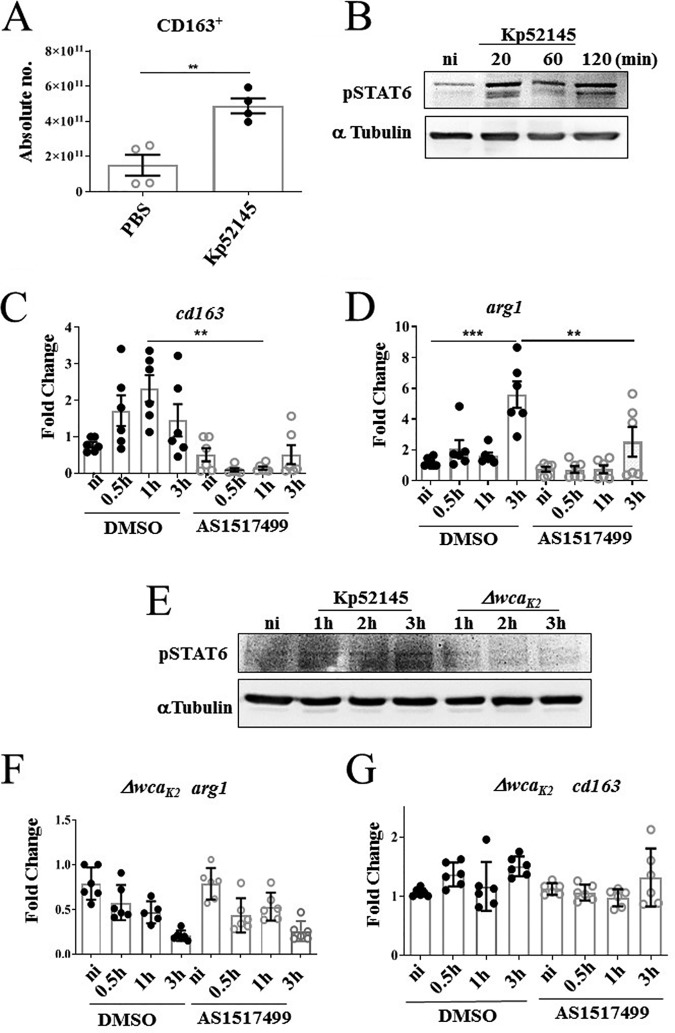
K. pneumoniae drives macrophage polarization in a STAT6-dependent manner. (A) CD163 surface expression in mock-infected (PBS) or Kp52145-infected pBMDMs by flow cytometry. Values are shown as the means ± SEM from two independent experiments in duplicate. **, *P* < 0.01, determined by unpaired Student’s *t* test. (B) Immunoblotting analysis of phosphorylation of STAT6 (PSTAT6) and tubulin in lysates of pBMDMs infected with Kp52145 for the indicated times or left noninfected (ni). Data are representative of those from three independent experiments. (C) *cd163* levels in pBMDMs left noninfected or infected with Kp52145 pretreated with STAT6 inhibitor (AS1517499, 50 nM, 2 h prior to infection) or DMSO vehicle control. Values are shown as the means ± SEM from three independent experiments. (D) Arginase-1 levels in pBMDMs left noninfected or infected with Kp52145 pretreated with STAT6 inhibitor (AS1517499, 50 nM, 2 h prior to infection) or DMSO vehicle control. Values are shown as the means ± SEM from three independent experiments. (E) Immunoblotting analysis of phosphorylation of STAT6 (PSTAT6) and tubulin in lysates of pBMDMs infected with Kp52145 and strain 52145-Δ*wca_K2_* for the indicated times or left noninfected. Data are representative of those from three independent experiments. (F) *cd163* levels in pBMDMs left noninfected or infected with strain 52145-Δ*wca_K2_* pretreated with STAT6 inhibitor (AS1517499, 50 nM 2 h prior to infection) or DMSO vehicle control. Values are shown as the means ± SEM from three independent experiments in duplicate. (G) *arginase-1* levels in pBMDMs left noninfected or infected with strain 52145-Δ*wca_K2_* pretreated with STAT6 inhibitor (AS1517499, 50 nM, 2 h prior to infection) or DMSO vehicle control. Values are shown as the means ± SEM from three independent experiments in duplicate. In panels C and D, significant difference was determined for the indicated comparisons using one-way ANOVA with Bonferroni correction. ***, *P* < 0.001; **, *P* < 0.01.

To further investigate whether K. pneumoniae governs macrophage polarization, we established a method to generate porcine bone marrow-derived macrophages (pBMDMs). We next sought to determine whether K. pneumoniae skews the polarization of pBMDMs. Infection of pBMDMs with Kp52145 resulted in a significant upregulation of the surface expression of the M2 marker CD163 as detected by flow cytometry (Fig. 6A). Furthermore, the analysis of the expression of several inflammation-associated cytokines and chemokines by RT-qPCR showed increased expression of the M2 marker *ccr1* in pBMDMs infected with Kp52145 (Fig. S3A). Kp52145 infection also upregulated the levels of *tnf-α* and *il-1β* (Fig. S3B and C). However, the levels induced by the *cps* mutant were significantly higher than those elicited by Kp52145 (Fig. S3B and C). Moreover, the levels of *il-12*, *il-6*, *ifn-γ*, *nos2*, and *il-8* were higher in macrophages infected with the *cps* mutant than in those infected with Kp52145, which were not significantly different than those PBS-mock infected (Fig. S3D to H). These results are in good agreement with those obtained infecting the porcine EVLP model. Collectively, the elevated levels of proinflammatory cytokines in macrophages infected with the *cps* mutant are consistent with M1 polarized macrophages, whereas the increased levels of CD163 and *ccr1* suggest that wild-type K. pneumoniae shifts the polarization of macrophages toward the M2 state.

10.1128/mBio.02802-19.3 FIG S3 K. pneumoniae CPS mutant induces M1 markers in pBMDMs. Shown are *ccr1*, *tnf-α*, *il-1β*, *il-12*, *il-6*, *ifn-γ*, *nos2*, and *il-8* levels in pBMDMs left noninfected (ni) or infected with K. pneumoniae 52.145 (Kp52145) and the isogenic *cps* mutant, strain 52145-Δ*wca_K2_* (Δ*wca_K2_*), for the indicated times. Values are shown as the means ± SEM from three independent experiments in duplicate. ****, *P* < 0.0001; ***, *P* < 0.001; **, *P* < 0.01; *, *P* < 0.05; n.s., *P* > 0.05 for the indicated comparisons using one-way ANOVA with Bonferroni correction. Download FIG S3, PDF file, 0.2 MB. Copyright © 2019 Dumigan et al. 2019 Dumigan et al. This content is distributed under the terms of the Creative Commons Attribution 4.0 International license.

STAT6 is a well-established transcription factor regulating M2 macrophage polarization (43, 44). Therefore, we sought to determine whether K. pneumoniae activates STAT6 to govern macrophage polarization in pBMDMs. Immunoblotting experiments revealed that Kp52145 induced the phosphorylation of STAT6 in pBMDMs (Fig. 6B). Phosphorylation of STAT6 is essential for its nuclear translation to control the transcription of STAT6-induced genes (45, 46). To establish whether *Klebsiella*-induced macrophage polarization is STAT6 dependent, we followed a pharmacological approach probing the STAT6 inhibitor AS1517499 (47). Transcriptional analysis showed that *Klebsiella*-induced expression of the M2 markers *cd163* and *arginase-1* (*arg-1*) was ablated in cells pretreated with the STAT6 inhibitor (Fig. 6C and D), demonstrating that *Klebsiella* induction of M2 markers is STAT6 dependent. Interestingly, and in agreement with our previous findings suggesting that the CPS could be required for *Klebsiella*-triggered macrophage polarization, the *cps* mutant did not induce the phosphorylation of STAT6 (Fig. 6E). As we anticipated, the *cps* mutant did not induce the expression of the M2 markers *arg-1* and *cd163* in pBMDMs (Fig. 6F and G).

**FIG 7:**
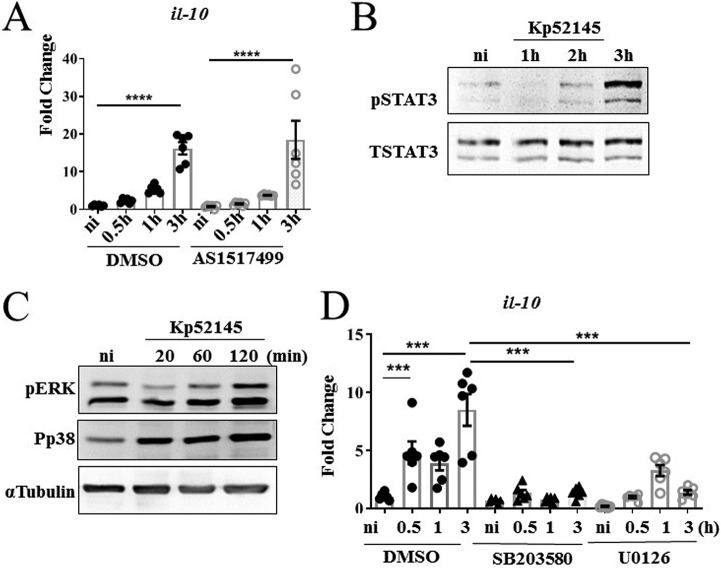
K. pneumoniae induces *il-10* expression, which is p38 and pERK dependent. (A) *il-10* levels in pBMDMs left noninfected or infected with Kp52145 pretreated with STAT6 inhibitor (AS1517499, 50 nM, 2 h prior to infection) or DMSO vehicle control. Values are shown as the means ± SEM from three independent experiments. (B) Immunoblotting analysis of phosphorylation of STAT3 (PSTAT3) and STAT3 in lysates of pBMDMs infected with Kp52145 for the indicated times or left noninfected. Data are representative of those from three independent experiments. (C) Immunoblotting analysis of phosphorylations of ERK (pERK), p38 (Pp38), and tubulin in lysates of pBMDMs infected with Kp52145 for the indicated times or left noninfected. Data are representative of those from three independent experiments. (D) *il-10* levels in pBMDMs left noninfected or infected with Kp52145 pretreated with p38 inhibitor (SB203580 [Tocris], 10 μg/ml, 2 h prior to infection), ERK inhibitor (U0126 [LC Laboratories], 20 μg/ml, 2 h prior to infection) or DMSO vehicle control. Values are shown as the means ± SEM from three independent experiments. In panels A and D, significant difference was determined for the indicated comparisons using one-way ANOVA with Bonferroni correction. ****, *P* < 0.0001; ***, *P* < 0.001.

Kp52145 also upregulated the transcription of the anti-inflammatory cytokine and M2 marker *il-10* in pBMDMs (Fig. 7A), indicating that the *il-10* expression observed in porcine EVLP tissues infected with Kp52145 (Fig. 5G) could be derived from macrophages. This increased expression was not dependent on STAT6 because the STAT6 inhibitor did not reduce the expression of *il-10* (Fig. 7A). In mouse and human macrophages, the transcription of *il-10* is regulated by STAT3 (48). Immunoblotting analysis confirmed the activation of STAT3 in *Klebsiella*-infected pBMDMs (Fig. 7B). Mitogen-activated protein (MAP) kinases p38 and extracellular signal-regulated kinase (ERK) are known to control the expression of IL-10 in mouse and human macrophages (48). Control experiments showed that Kp52145 infection induced the phosphorylation of p38 and ERK MAP kinases in pBMDMs (Fig. 7C). As we anticipated, pharmacological inhibition of p38 and ERK with SB203580 and U0126, respectively, resulted in decreased expression of *il-10* in infected pBMDMs (Fig. 7D).

Together, these results demonstrate that K. pneumoniae skews macrophage polarization toward an M2 state in an STAT6-dependent manner. Furthermore, our results indicate that *Klebsiella*-induced macrophage polarization is dependent on the CPS.

## DISCUSSION

The development of infection models that approximate human disease is essential not only for understanding pathogenesis at the molecular level but also to test new therapies before entering into clinical stages. This is particularly relevant given the costs of clinical trials and the impact on the health system. Animal models, chiefly rodents, have provided invaluable information, and not surprisingly, they are used in most of the preclinical studies. However, the limitations of these models in terms of yielding accurate preclinical data to inform clinical trials are widely recognized. Furthermore, in the context of infectious diseases it is an established fact that there are significant differences between rodents, mice, and humans in terms of immune activation following infection (2). In fact, the different immune/inflammatory pathways existing between mice and humans have fueled the conclusion that murine studies are unreliable predictors of human outcomes. In this regard, porcine models are becoming increasingly important as ideal preclinical models. The anatomical and physiological similarities between pigs and humans, including the activation of the immune system, argue in favor of using pigs to model human diseases.

The results of this study strongly suggest that the EVLP model using pig lungs could be considered a platform to investigate the infection biology of respiratory pathogens and, eventually, to run preclinical studies testing new therapeutics. As a proof of principle, we provide evidence demonstrating that infection of the porcine EVLP with the human pathogen K. pneumoniae recapitulates the known features of *Klebsiella*-triggered pneumonia, including the lung injury associated with the infection and the recruitment of neutrophils and other immune cells following infection (9). Moreover, our data revealed that the EVLP model is useful to assess the virulence potential of K. pneumoniae. The K. pneumoniae *cps* mutant previously known to be attenuated in other models (24, 25, 28, 29) was also attenuated in the porcine EVLP model.

To set up the EVLP infection model using pig lungs, we took advantage of the advances in organ preparation for lung transplants. The EVLP method has become prevalent in lung transplant centers around the world (20) and has been proven as a means to prolong the window for transplant evaluation (49). The lung transplant community has developed a robust protocol for EVLP that can capture key physiologic parameters (gas exchange, lung mechanics, pulmonary vascular hemodynamics, and edema) and to obtain samples for limited analysis (20). In our study, we have adapted the EVLP technology used for human lungs to pig lungs, and we have developed a robust infection method to assess the infection biology of respiratory pathogens. Although in this study we have focused on K. pneumoniae, the model is amenable to use with other bacterial pathogens but also viruses and fungi. To set up the model for other pathogens, scientists will need to consider the pathogen inoculum and the time of infection, which might go up to 24 h. The quality of the organ is a crucial aspect when considering long infection times.

*Ex vivo* modeling is superior to tissue- and cell-based assays because the architectural integrity of the lung is preserved. For example, type I pneumocytes, which cover over 90% of the gas exchange surface of the lung, are difficult to culture *in vitro*; therefore, little is known about the response of this cell type to injury and infection. Our model is a significant step change from the previous elegant infection model using *ex vivo* sections of pig lungs to assess bacterial virulence (50). This cell-free model allows investigation of pathogen physiology, including transcriptomics, in a spatially structured environment. Furthermore, the model allows a medium-throughput analysis of pathogen virulence factors. However, the porcine EVLP model developed in this study facilitates the study of the functional interactions between different immune cells, dendritic cells, neutrophils, macrophages, and epithelial cells in a more physiological setting. Additionally, we believe that our model is more suitable to test disease-modifying therapies in acute lung injury to generate relevant, reliable, and predictable human pharmacodynamic, pharmacokinetic, and toxicology data through analysis at the organ (51). The main advantage of using pig versus human lungs is the availability of the former. Human lungs not suitable for transplant are scarce, and the access to them is expensive. Recently, we have successfully adapted the EVLP model using human lungs to study *Klebsiella* infection biology.

Another novel finding of our study is that *Klebsiella* skews macrophage polarization to an M2-like state. Importantly, our findings uncovered that *Klebsiella*-induced macrophage polarization is dependent on the activation of STAT6, the most important transcriptional factor governing M2 polarization (43, 44). M1 phenotype is characterized by the expression of high levels of proinflammatory cytokines, high production of reactive oxygen intermediates and inducible nitric oxide synthase (iNOS)-dependent reactive nitrogen intermediates, promotion of the Th1 response by interleukin 12 (IL-12) production, and potent microbicidal activity (35, 42). In contrast, M2 macrophages are characterized by the selective expression of markers such as arginase 1 and CD163 as well as the production of low levels of IL-12 and iNOS and enhanced IL-10 production (35, 42). It should be noted that the classification of macrophages into M1 and M2 cells is a simplified descriptor of the plasticity of these cells. M1 and M2 functional phenotypes represent two extremes of a spectrum of possible states of macrophage activation during homeostasis and inflammation. Macrophage polarization is driven by signals in the tissue microenvironment, including cytokines and pathogens. These cues dictate a transcriptional response shaping the function of the macrophages, but always on the basis of a pathophysiological context. M1 macrophages are generally considered responsible for resistance against intracellular pathogens (34). Not surprisingly, a growing number of studies show that some pathogens have evolved different strategies to interfere with M1 polarization (34), whereas there are few examples of intracellular pathogens (*Francisella*, *Salmonella*, *Coxiella*, and *Tropheryma*) inducing an anti-inflammatory M2 state (52–55). The potential impact of *Klebsiella* on macrophage plasticity has been largely overlooked. Most likely this is due to the fact that *Klebsiella* has been traditionally considered an extracellular pathogen, although our laboratory has recently demonstrated that *Klebsiella* survives intracellularly in mouse and human macrophages by preventing phagolysosome fusion (19). We believe that shifting macrophage polarization toward an M2-like state is a widespread immune evasion strategy exploited by bacterial pathogens to survive in tissues. Future studies are warranted to validate this notion investigating the growing list of pathogens able to counteract the antimicrobial action of macrophages.

We were keen to identify *Klebsiella* factors involved in modulating macrophage plasticity. This is even more relevant because *Klebsiella* does not encode either a type III or a type IV secretion system employed by intracellular pathogens to target macrophage polarization. The facts that the attenuated *cps* mutant did not activate STAT6 and did not induce an M2-like state strongly suggest that the induction of an M2-like state is a virulence strategy of *Klebsiella* to promote infection. We and others have provided compelling evidence showing that K. pneumoniae CPS is a bona fide immune evasin (15, 18, 25, 56–59). The results of this study further reinforce this notion by demonstrating that the CPS skews macrophage polarization toward an M2 state. Further studies are warranted to investigate whether this could be a general feature of other CPSs.

Interestingly, our findings provide an explanation for the clinical observation that some health factors, such as alcohol abuse or viral infections, are associated with increased susceptibility to *Klebsiella* infections (60–62). These factors are known to increase the number of M2 macrophages in the lung (63–65), which then could facilitate *Klebsiella* infection. Supporting this hypothesis, there is an improvement in bacterial clearance when this macrophage population is eliminated *in vivo* (63–65).

Despite the clear utility of the EVLP model to assess infections, it is worthwhile commenting on the limitations. The process recapitulated in the EVLP model represents early steps in the infection process and does not model other aspects such as organ dissemination. In addition, the model does not integrate other signals, such as those from the gut, known to be relevant to control infections (66–68). Further impediments are the difficulties to generate cell-specific knock-in or knockouts and the relatively low throughput of the model to test several bacterial mutants. However, we believe that the advantages significantly outweigh the limitations, and the EVLP model is a useful translational preclinical model to illuminate new aspects of the infection biology of pathogens such as those identified in this work.

## MATERIALS AND METHODS

### Collection of lungs and whole blood.

Lungs were collected as part of the by-product of other studies. Lungs could be obtained also from abattoirs, although researchers should be on-site to assess the quality of the lungs and collect the organ and the whole blood. In our case, immediately after euthanization, 200 ml of whole blood was collected rapidly in sterile receptacles containing 10% citrate-phosphate-dextrose solution (C7165; Sigma), an anticoagulant; whole blood was then mixed gently and kept at room temperature. Both lungs and heart were promptly removed by sharp dissection. The heart was removed, leaving an ample (>3 cm) section of pulmonary artery intact. Lungs were then separated along the carina, again leaving at least 3 cm of trachea intact. As pigs have an additional bronchus (cranial) on the right lung, only left lungs were selected for this study, as they are readily compatible with the LS1 system and are anatomically more similar to human lungs. The left lung was then flushed gradually with 500 ml of Dulbecco’s modified Eagle medium (DMEM) without phenol red via the pulmonary artery to remove blood using a 50-ml syringe. Tissue was then wrapped in plastic and placed on ice for transportation to the lab. Lungs were rejected for this study if they contained large areas of hemorrhage or consolidation.

### Preparation of lungs for EVLP.

A cannula was placed in pulmonary artery and connected to the efferent tube of the VivoLine LS1 reconditioning unit to facilitate perfusion. Similarly, an endobronchial tube was inserted into the bronchus and secured with sutures before being connected to a ventilator circuit with adult bacterial viral filters (140141/1; DS Medical). The LS1 temperature probe was placed in pulmonary veins and secured in place using a surgical suture. A perfusate consisting of 2 liters of DMEM (Invitrogen) without phenol red and supplemented with 5% l-glutamine and 5% fetal calf serum (FCS) was placed in the base of the reservoir. The target temperature was set to 37°C. Initial perfusion began with 0.05 liter/min, at this point ensuring that the LS1 shunt is open, and flow gas gradually increased to 0.4 liter/min, maintaining a pulmonary artery pressure of 10 to 15 mm Hg. Once a temperature of 30°C was reached, the lungs were gently inflated with an Ambu bag. Ensuring that the lung is warm prior to inflation reduces the risk of capillary damage. Continuous positive airway pressure (CPAP) of 10 cm H_2_O was applied with 95%O_2_–5% CO_2_ using a mechanical ventilator (Dräger Evita). Once the system reached 36°C with the desired pressure, 200 ml of autologous blood was added to the perfusate to act as a reservoir for immune cell recruitment.

### Bronchoalveolar lavage.

Once a temperature of 36°C was reached, a baseline bronchoalveolar lavage (BAL) sample was collected by inserting a catheter (PE 240-Harvard apparatus) into the subsegment (caudal) lobe via the endotracheal tube and gently advanced until resistance was encountered, at which point the catheter was withdrawn by 1 cm. Then 125 ml of warmed normal saline was instilled and retrieved after 5 min through the same catheter. The catheter was then used to deliver 5 ml of sterile PBS or 5 × 10^5^ CFU of Kp52145 in 5 ml of PBS. After 4 h, BAL sampling was repeated prior to disconnecting the lung and tissue collection was carried out. BAL samples were assessed for total protein and innate immune infiltrates.

### Preparation of bacteria.

K. pneumoniae 52.145, a clinical isolate (serotype O1:K2) previously described (22, 69), was utilized alongside the isogenic *cps* mutant, strain 52145-Δ*wca_K2_*, which has been described previously (70). Bacteria were tagged with GFP by transformation with plasmid pFPV25.1 Cm (25). For infections, a single colony was cultured in 5 ml of LB broth overnight at 36°C with gentle agitation. After 1:10 dilution, bacteria were grown to exponential phase by incubation at 37°C with agitation for 2.5 h. Bacteria were then adjusted an optical density at 600 nm (OD_600_) of 1.0 in PBS. For *in vitro* infections, macrophages were infected with a multiplicity of infection (MOI) of 100:1, whereas 5 × 10^5^ CFU/ml were used to infect the lungs. CFU in the tissue were determined by homogenizing 100 μg of tissue from the caudal lobe in 1 ml of sterile PBS and plating serial dilutions on salmonella-shigella agar plates (Sigma). Three samples were assessed across each lung. Plates were incubated overnight at 37°C before counting. When required, antibiotics were added to the growth medium at the following concentrations: rifampin, 50 μg/ml, and chloramphenicol, 25 μg/ml.

### Protein quantification.

Protein concentration was assessed in BAL samples at baseline (0 h) and at 4 h. Standards and samples were incubated with the Pierce 660-nm protein assay (150 μl of reagent to 10 μl of sample/standard) at room temperature for 5 min prior to quantification using a NanoDrop spectrophotometer as per manufacturer instructions (Thermo Scientific).

### Histology.

Tissue sections (∼1 cm^3^) were collected from the cranial, middle, and caudal lobes of each lung fixed in 10 ml 10% formalin in a 15-ml Falcon tube with an inverted p1000 tip to submerge tissue. After a minimum of 48 h at room temperature, samples were processed for paraffin embedding, sectioning, and hematoxylin and eosin staining. Samples were imaged using a DM5500 Leica vertical microscope at a magnification of ×200. Alveolar septal edema was quantified by measuring alveolar septal thickness with ImageJ software, whereby three measurements of the thickest septa were acquired per image and averaged and 30 images were acquired, whereby 10 images were acquired per section and 3 sections per lung. Alveolar septa adjacent to a blood vessel or airway were excluded due to normal thickening resulting from collagen deposition. Intra-alveolar hemorrhage and the presence of intra-alveolar mononuclear cells and proteinaceous debris were also recorded. Histological scores were assigned based on parameters set by Matute-Bello and coworkers (30). Hemorrhage was scored as follows: 0, none; 1, mild; 2, moderate; and 3, severe. Proteinaceous debris scored as follows: 0, none; 1, protein present; and 2, abundant presence of protein in alveolar spaces. The number of nucleated cells within the alveolar space was counted and presented as intra-alveolar leukocytes. Five images were scored per section, with 3 sections per lung at a magnification of ×400.

### RNA purification.

A total of 100 μg of lung tissue was homogenized using a VDI 12 tissue homogenizer (VWR) in 1 ml of TRIzol reagent (Ambion) and incubated at room temperature for 5 min before being stored at –80°C. RNA was extracted from pBMDMs using an RNeasy minikit (Qiagen reference no. 74104). Total RNA was extracted according to the manufacturer’s instructions. A total of 5 μg of total RNA was treated with recombinant DNase I (Roche Diagnostics Ltd.) at 37°C for 30 min and then purified using a standard phenol-chloroform method. The RNA was precipitated with 20 μl of 3 M sodium acetate (pH 5.2) and 600 μl of 98% (vol/vol) ethanol at –20°C, washed twice in 75% (vol/vol) ethanol, dried, and then resuspended in RNase-free H_2_O. Duplicate cDNA preparations from each sample were generated from 1 μg of RNA using Moloney murine leukemia virus (M-MLV) reverse transcriptase (Sigma-Aldrich) according to the manufacturer’s instructions. RT-qPCR analysis of cytokine-related porcine gene expression was performed using the KAPA SYBR-FAST qPCR kit (KAPA Biosystems), using the primers shown in Table 1. Samples were run using the Stratagene Mx3005P qPCR system (Agilent Technologies). Nontemplate negative controls to check for primer-dimer and a porcine genomic DNA were included. Thermal cycling conditions were as follows: 95°C for 3 min for enzyme activation, 40 cycles of denaturation at 95°C for 10 s, and annealing at 60°C for 20 s. cDNA samples were tested in duplicate and relative mRNA quantity was determined by the comparative threshold cycle (ΔΔ*C_T_*) method using the hypoxanthine phosphoribosyltransferase (HPRT) housekeeping gene for normalization.

**TABLE 1:** List of primers used in this study for RT-qPCR

Gene	Primer sequence
*il-6*	Forward, 5′-GACAAAGCCACCACCCCTAA-3′; reverse, 5′-CTCGTTCTGTGACTGCAGCTTATC-3′
*il-1β*	Forward, 5′-GAGCATCAGGCAGATGGTGT-3′; reverse, 5′-CAAGGATGATGGGCTCTTCTTC-3′
*tnf-α*	Forward, 5′-GGCCCAAGGACTCAGATCAT-3′; reverse, 5′-CTGTCCCTCGGCTTTGACAT-3′
*ccr1*	Forward, 5′-GTGCTGCCTCTATTGGTCAT-3′; reverse, 5′-ACCTCTGTCACTTGTATGGC-3′
*il-12*	Forward, 5′-CGTGCCTCGGGCAATTATA-3′; reverse, 5′-CGCAGGTGAGGTCGCTAGTT-3′
*il-8*	Forward, 5′-ATGACTTCCAAACTGGCTG-3′; reverse, 5′-CTTGTTGTTGTTACTGCTG-3′
*ifn-γ*	Forward, 5′-CTCTCCGAAACAATGAGTTATACAA-3′; reverse, 5′-GCTCTCTGGCCTTGGA-3′
*nos2*	Forward, 5′-CCACCAGACGAGCTTCTACC-3′; reverse, 5′-TCCTTTGTTACCGCTTCCAC-3′
*stat-4*	Forward, 5′-GAAAGCCACCTTGGAGGAAT-3′; reverse, 5′-ACAACCGGCCTTTGTTGTAG-3′
*il-10*	Forward, 5′-GCCTTCGGCCCAGTGAA-3′; reverse, 5′-AGAGACCCGGTCAGCAACAA-3′
*cd163*	Forward, 5′-CCAGTGAGGGAACTGGACAC-3′; reverse, 5′-GGCTGCCTCCACCTTTAAGT-3′
*arginase-1*	Forward, 5′-AGAAGAACGGAAGGACCAGC-3′; reverse, 5′-CAGATAGGCAGGGAGTCAC-3′
*hprt*	Forward, 5′-ACACTGGCAAAACAATGCAA-3′; reverse, 5′-ACACTTCGAGGGGTCCTTTT-3′

### Flow cytometry.

A total of 100 μg of lung tissue was homogenized in 1 ml of sterile PBS and filtered through a 70-μm cell strainer (2236348; Fisherbrand). Cells were centrifuged and red blood cells lysed using ammonium chloride-potassium lysis buffer (A1049201; Gibco) for 3 min at room temperature and washed with 1 ml of PBS prior to staining with the following mouse anti-pig antibodies: CD11R3 (MCA2309), CD163 (clone 2A10), SLA class II, and granulocyte antibody (clone 6D10) (AbD Serotech). Each purified anti-pig antibody was labeled with a fluorophore using Abcam phycoerythrin (PE; ab102918), allophycocyanin (APC)-Cy5.5 (ab102855), fluorescein isothiocyanate (FITC; ab102884), and rhodamine (ab188286) conjugation kits.

### Generation of porcine bone marrow-derived macrophages (pBMDMs).

Femurs from pigs between 80 and 100 kg were cleared of all muscle and sinew. Bone was then washed with 70% ethanol. A sterilized junior hacksaw was used to cut transversely across bone to expose bone marrow under sterile conditions. A total of 5 g of bone marrow per 50-ml tube was suspended in 40 ml of complete medium and centrifuged at 600 × *g* for 8 min to remove fat. Red blood cells were lysed via incubation with ammonium-chloride-potassium lysis buffer (A1049201; Gibco) for 3 min. Cells were washed in 10 ml of complete medium and passed through a 70-mm cell strainer (2236348; Fisherbrand) prior to centrifugation. Cell pellet was dislodged before plating on 20-cm petri dishes (Sarstedt) in 25 ml of complete medium (DMEM, high glucose, GlutaMAX, supplemented with 10% FCS and 1% penicillin-streptomycin) and 5 ml of syringe-filtered L929 supernatant (a source of macrophage colony-stimulating factor [M-CSF]). Cells were cultured for 6 days before assessment of purity by flow cytometry.

### *In vitro* infections.

pBMDMs were seeded in 6-well dishes (5 × 10^5^ cells/well) in complete medium (DMEM, high glucose, GlutaMAX, supplemented with 10% FCS and 1% penicillin-streptomycin) and allowed to adhere overnight. Complete medium was removed and replaced with antibiotic-free medium prior to infection. The bacterial inoculum was prepared as previously indicated, and cells were infected with an MOI of 100 bacteria per cell. To synchronize infection, plates were centrifuged at 200 × *g* for 5 min. After 1 h, medium was removed and replaced with antibiotic-free medium supplemented with 100 μg/ml of gentamicin (Sigma) to kill extracellular bacteria. For STAT6 inhibition, cells were serum starved and incubated with the chemical STAT6 inhibitor AS1517499 (50 nM, 919486-40-1; AXON Medchem) or dimethyl sulfoxide (DMSO) as a vehicle control for 2 h prior to infection and maintained throughout. To inhibit pERK and p38 activity, the chemical inhibitors U0126 (20 μg/ml; LC Laboratories) and SB203580 (10 μg/ml; Tocris) were utilized, respectively, 2 h prior to infection and maintained throughout the experiment. At the desired time points, supernatants were removed and cells lysed for analysis by Western blotting or RT-qPCR.

### Western blotting.

At an appropriate time point postinfection, cells were washed with ice-cold PBS before lysis in Laemmli buffer (4% SDS, 10% 2-mercaptoehtanol, 20% glycerol, 0.004% bromophenol blue, 0.125 M Tris-HCl [pH 6.8]). Lysates were sonicated for 10 s at 10% amplitude, boiled at 95°C for 5 min, and centrifuged at 12,000 × *g* for 1 min prior to running on 8% SDS-PAGE. Samples were transferred onto a 0.2-mm nitrocellulose membrane (Biotrace; VWR) using a semidry transfer unit (Bio-Rad) before blocking nonspecific antibody binding for 1 h in 3% bovine serum albumin (BSA) in Tris-buffered saline (TBS) with 1% Tween 20. Primary antibodies included phospho-STAT6 (Tyr641) (1:2,000, no. 9361), phospho-STAT3 (Y705) (1:2,000, no. 9145), total STAT3 (1:2,000, no. 12640), phospho-ERK (p44/42) (1:2,000, no. 91015), and phospho-p38 (T180/Y182) (1:2,000, no. 4511), all from Cell Signaling Technologies. Blots were incubated with appropriate horseradish peroxidase-conjugated secondary antibody goat anti-rabbit immunoglobulins (1:5,000, no. 170-6515; Bio-Rad) or goat anti-mouse immunoglobulins (1:1,000, no. 170-6516; Bio-Rad). Protein bands were detected using chemiluminescence reagents and a G:BOX Chemi XRQ chemiluminescence imager (Syngene). To detect multiple proteins, membranes were reprobed after stripping of previously used antibodies using a pH 2.2 glycine HCl–SDS buffer. To ensure that equal amounts of proteins were loaded, blots were reprobed with α-tubulin (1:2,000, no. 2125; Cell Signaling Technologies).

### Statistics.

Statistical analyses were performed with Prism 6 (GraphPad Software) using 1-way analysis of variance (ANOVA) with Bonferroni correction or unpaired two-tailed Student’s *t* test. Error bars indicate standard errors of the means (SEM). Statistical significance is indicated in figures as follows, unless otherwise specified: ns, not significant (*P* > 0.05); *, *P* < 0.05; **, *P* < 0.01; ***, *P* < 0.001; and ****, *P* < 0.0001.
