# Supplementary figures and images for: A Porcine Ex Vivo Lung Perfusion Model To Investigate Bacterial Pathogenesis
Source: mBio. 2019 Dec 3;10(6):e02802-19. doi: 10.1128/mBio.02802-19 (PMC6890995; doi:10.1128/mBio.02802-19)

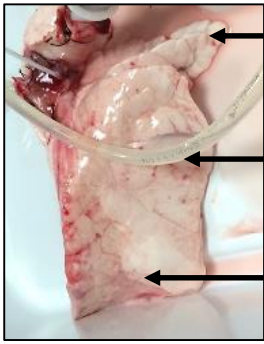

**Cranial**

**Middle**

**Caudal**

Supplement: FIG S1 [file mBio.02802-19-sf001.pdf]

**A**

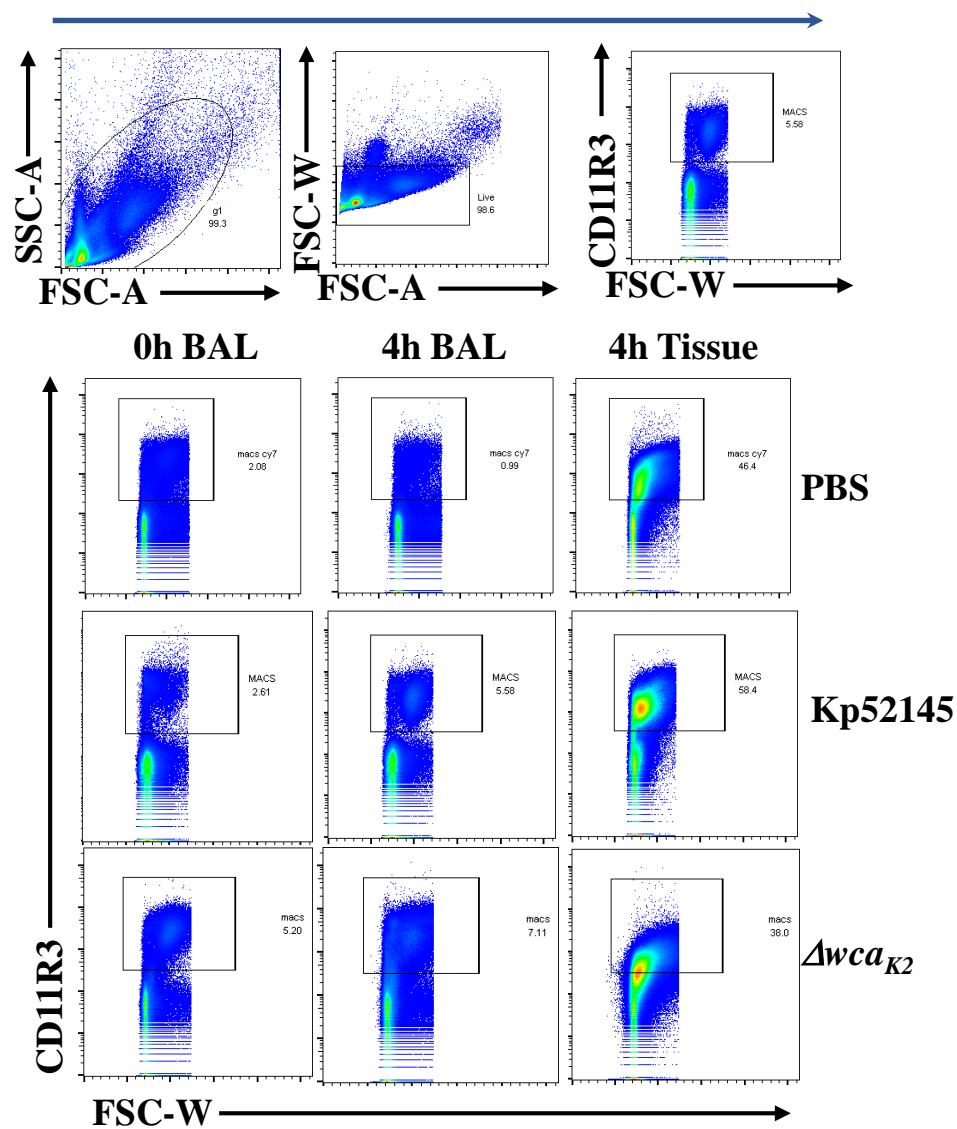

**B**

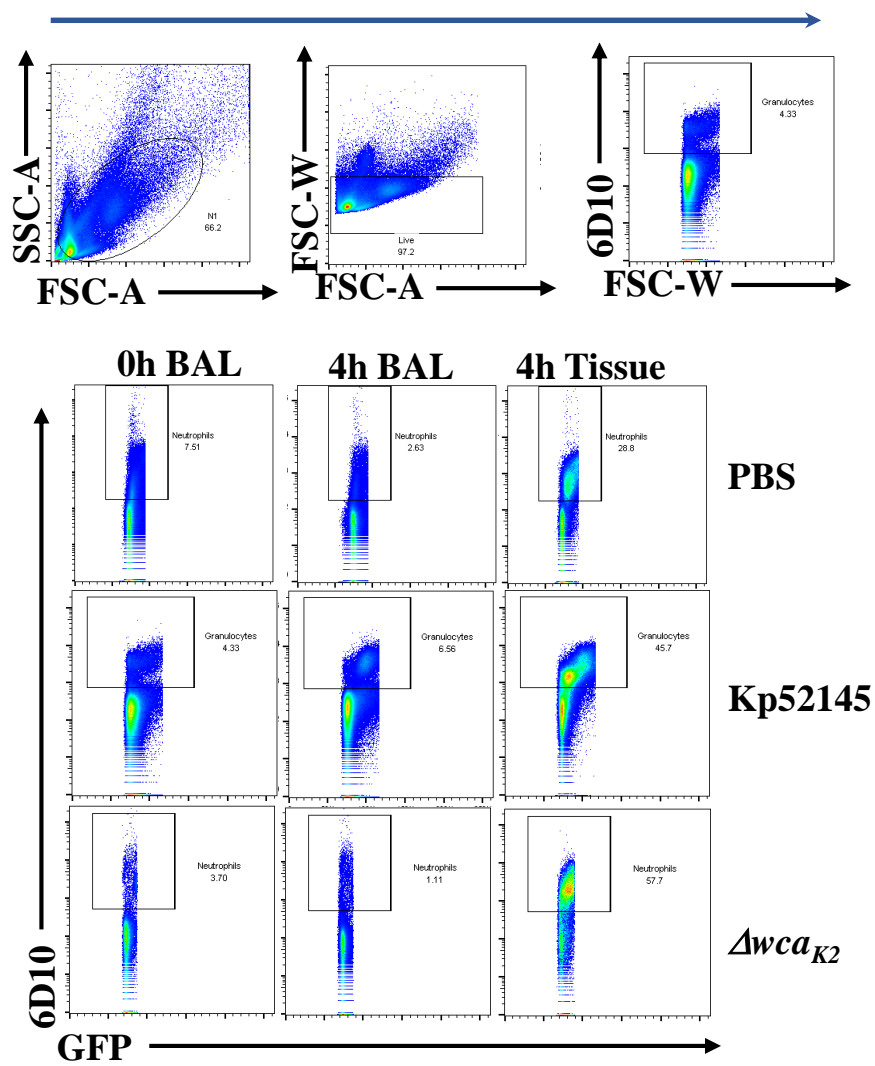

C

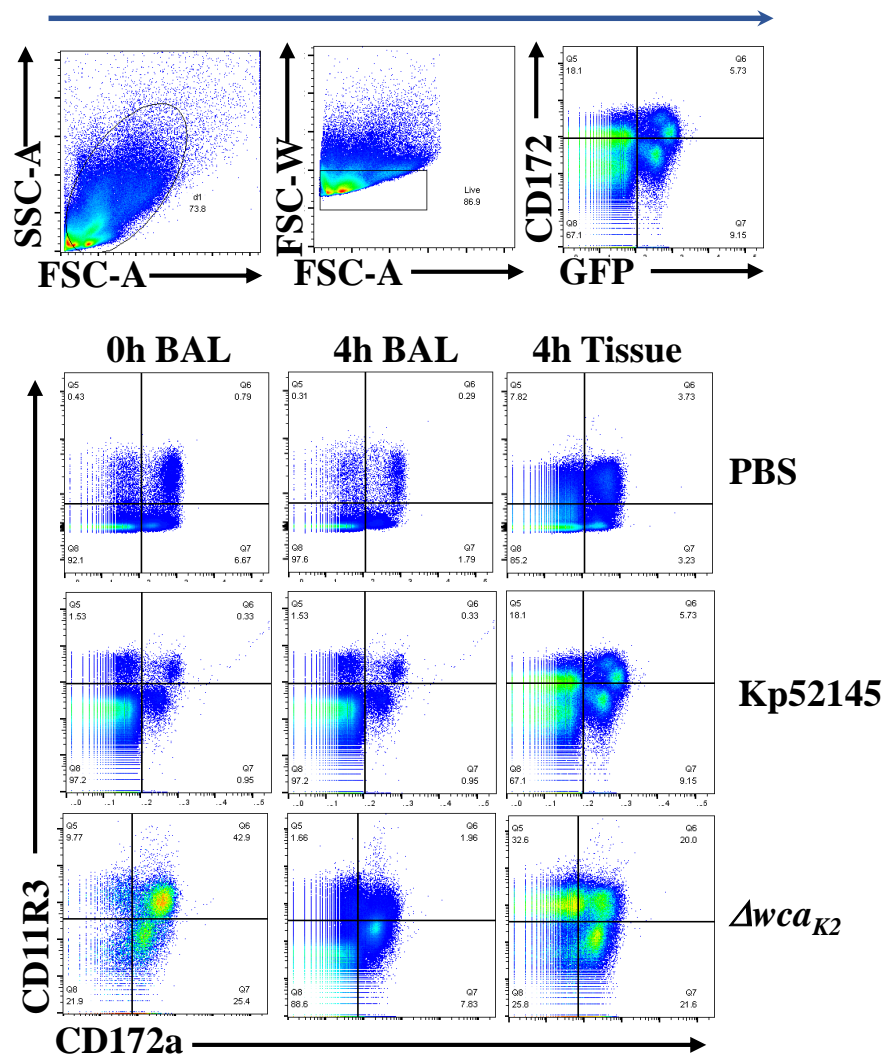

D

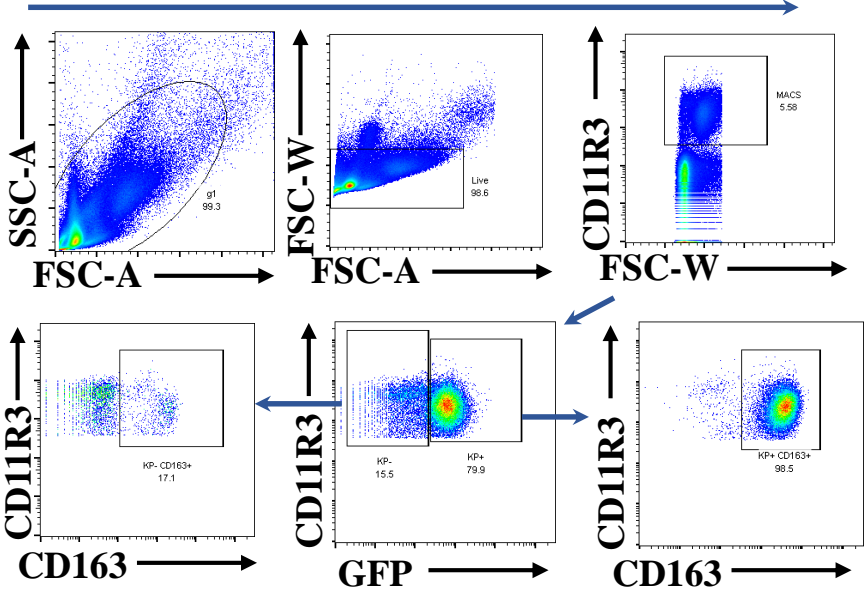

Supplement: FIG S2 [file mBio.02802-19-sf002.pdf]

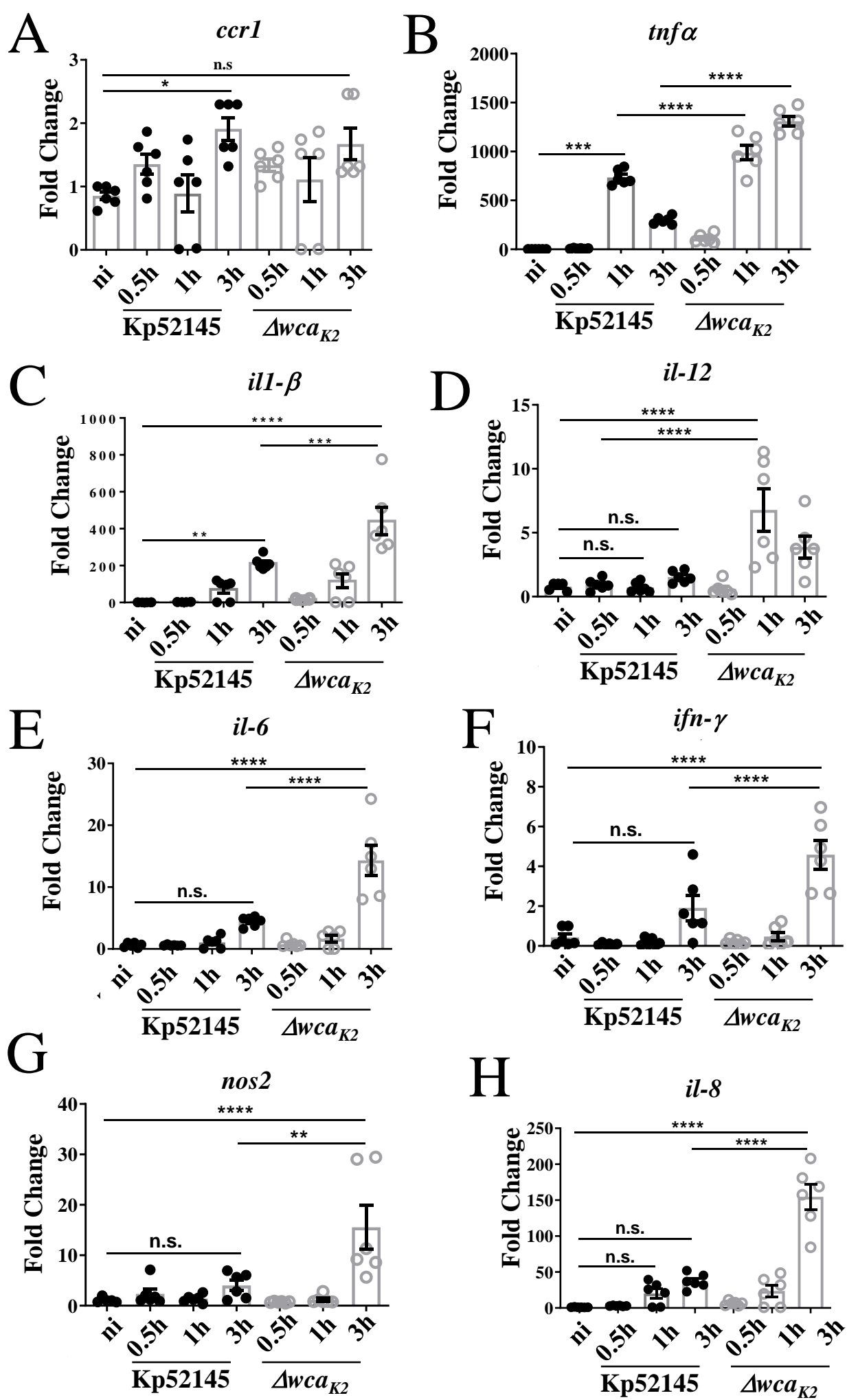

Supplement: FIG S3 [file mBio.02802-19-sf003.pdf]
